# Resistance Patterns and Phenotypic Detection of β-lactamase Enzymes among Enterobacteriaceae Isolates from Referral Hospitals in Khartoum State, Sudan

**DOI:** 10.7759/cureus.7260

**Published:** 2020-03-13

**Authors:** Maha Dirar, Naser Bilal, Mutasim E Ibrahim, Mohamed Hamid

**Affiliations:** 1 Medical Microbiology, Faculty of Medical Laboratory Sciences, University of Khartoum, Khartoum, SDN; 2 Basic Medical Sciences (Microbiology Unit), University of Bisha, Bisha, SAU; 3 Clinical Microbiology and Parasitology, King Khalid University, Abha, SAU

**Keywords:** enterobacteriaceae, resistance mechanisms, beta-lactamases, phenotypic detection, sudan

## Abstract

Background

Beta-lactamase enzymes-producing Enterobacteriaceae have emerged in many hospital settings resulting in poor treatment outcomes. We aimed to determine resistant patterns of Beta-lactamase enzymes among Enterobacteriaceae collected from referral hospitals in Khartoum state, Sudan.

Methods

A total of 168 Enterobacteriaceae recovered from clinical samples of patients during May 2014 to February 2015. Identification and susceptibility testing of the isolates were performed as per standard methods. Double-disk synergy test was applied to determine the presence of extended-spectrum β-lactamase (ESBL) production. AmpC beta-lactamases and carbapenemase were screened using AmpC disk test and the modified Hodge test, respectively.

Results

ESBL-producing Enterobacteriaceae represented 45.2%, with a higher rate among K. pneumoniae. AmpC beta-lactamase detected as 49.3%, with peak levels among Acinetobacter baumannii (A. baumannii) (83.3%) and Enterobacter cloacae (75%). Carbapenemase production was found among 74.5% of isolates, with high rates among A. baumannii (89%) and K. pneumoniae (78%). Overall Enterobacteriaceae, highest resistance rates were found in penicillins and cephalosporins agents. Amikacin and imipenem revealed good activities against most of the isolates, except for A. baumannii (66.7% and 75%, respectively). E. coli yielded high resistance rates for amoxicillin (98.8%), amoxicillin-clavulanic acid (93.8%), cefotaxime (93.8%), and ciprofloxacin (76.5%). Moderate resistance rates were observed among K. pneumoniae for ciprofloxacin (61.5%), nitrofurantoin (57.7%) and cefoxitin (40.4%).

Conclusions

ESBL, AmpC beta-lactamase and carbapenemase-producing Enterobacteriaceae are emerging and may contribute to increasing antimicrobial resistance in Sudan. Phenotypic screening of such enzymes is rapid and straightforward and should be simultaneously done and carried out routinely in our hospitals.

## Introduction

Members of Enterobacteriaceae continue to be an essential cause of several healthcare-associated infections [1]. Antimicrobial resistance among these Gram-negative rods is increasing on a worldwide basis, especially resistance against β-lactam agents due to the development of β-lactamase enzymes [2]. In the recent years, β-lactamase-producing Enterobacteriaceae are accountable for various health-associated infections, leading to higher mortality rates and medical costs than non-β-lactamase-producers [3]. There are different types of mechanisms by which Enterobacteriaceae can confer resistance to wide range of antimicrobial agents including, penicillins, cephalosporins, carbapenems and aztreonam but are inhibited by clavulanic acid [3,4]. However, β-lactamase-producing Enterobacteriaceae are commonly cross-resistant to other classes of antibiotics, such as fluoroquinolones, trimethoprim/sulfamethoxazole, and aminoglycosides, which results in limited therapeutic options to treat infections caused by these pathogens [3]. Extended-spectrum β-lactamase (ESBL) and AmpC β-lactamase are important mechanisms of beta-lactam resistance among Enterobacteriaceae [5]. Carbapenemases are β-lactamases and able to hydrolize β-lactam antibiotics, including carbapenems and also become a significant resistance mechanism in Enterobacteriaceae [6]. These β-lactamase enzymes-producing Enterobacteriaceae have emerged in many hospital settings making the choice of antibiotic treatment of infections by this bacterium very limited [7].

Increasing rates of β-lactamase-producing microorganisms are widely reported in many parts of the world [2,3,8]. However, early diagnosis followed by appropriate treatment is essential to decrease both mortality and morbidity rates of diseases caused by such pathogenic bacteria. Several phenotypic methods have been recommended for β-lactams detection on isolated Enterobacteriaceae strains [1,9]. The double-disk synergy test (DDST), the combination disk method, and the ESBL E test have been developed [1]. The modified Hodge test (MHT) is recommended by the Clinical and Laboratory Standards Institute (CLSI) as a carbapenemase-screening method [10]. It is well known that the AmpC disk test is an accurate tool for the detection of plasmid-mediated AmpC β-lactamases in bacteria lacking a chromosomally mediated AmpC β-lactamase [11]. This study aimed to determine the resistant patterns and phenotypic screening of β-lactamase enzymes among various clinical Enterobacteriaceae collected from referral hospitals in Khartoum state, Sudan.

## Materials and methods

Bacterial isolates and antimicrobial susceptibility testing

A total of 168 non-repetitive clinical Enterobacteriaceae were collected during a period from May 2014 to February 2015 from microbiology laboratories of five referral hospitals in Khartoum state, Sudan. It was a laboratory study and did not involve any human subject; therefore, ethical clearance was not necessary as per study guidelines. The isolates were obtained from the various clinical specimens submitted to the microbiology laboratories of Khartoum Teaching Hospital, Bahri Teaching Hospital, Soba University Hospital, Omdurman Teaching Hospital and Sharg Alnil Hospital during a routine investigation of possible bacterial pathogens. Each laboratory used their standard protocol for the isolation and identification of Enterobacteriaceae. Then the BD Phoenix (Becton Dickinson, Franklin Lakes, NJ) was used to confirm the identity of the collected isolates using Gram-negative phoenix panel according to Manual Instructions.

Antimicrobial susceptibility testing of the isolates was performed by the Kirby-Bauer disk diffusion assay on Mueller-Hinton agar medium against 14 antibacterial agents following the CLSI guidelines [12]. The antimicrobial agents (Oxoid, England) tested include: amikacin (30 μg), amoxicillin (10 μg), amoxicillin-clavulanic acid (30 μg), cefotaxime (30 μg), cefoxitin (30 μg), ceftazidime (30 μg), ceftriaxone (30 μg), cefuroxime (30 μg), ciprofloxacin (5 μg), gentamicin (10 μg), imipenem (10 μg), nitrofurantoin (50 μg), ofloxacin (5 μg) and trimethoprim/sulfamethoxazole (25 μg).

Screening of β-lactamases enzymes

DDST for ESBL Production

The DDST test was achieved as previously described [13]. This test was carried out immediately along with susceptibility testing for each isolate. A susceptibility disk containing amoxicillin-clavulanic acid was placed in the center of the plate, and disks containing ceftazidime and cefotaxime were placed 30 mm (center to center) from the amoxicillin-clavulanic acid disk. ESBL producing isolate was indicated by an extension of the edge of the inhibition zone of ceftazidime and/or cefotaxime towards amoxicillin-clavulanic acid disk after 48 hours incubation at 37°C aerobically.

AmpC Disk Test

Any isolates yielded a cefoxitin zone diameter less than 18 mm, and resistant to the third generation cephalosporins were tested for AmpC enzyme production by AmpC disk test. An exactly 0.5 McFarland suspension of E. coli ATCC 25922 was inoculated on the surface of Mueller-Hinton agar plate. A 30 μg cefoxitin disc was placed on the inoculated surface of the agar. A sterile plain disk was inoculated with several colonies of the test organism and placed beside the disk of cefoxitin, almost touching it, with the inoculated disk face in contact with the agar surface. After overnight incubation at 37°C, an indentation or a flattening of the zone of inhibition on the plates indicated enzymatic inactivation of cefoxitin was considered as a positive result. The absence of distortion stated no significant inactivation of cefoxitin and reported as a negative finding.

The Modified Hodge Test for Carbapenemase Production

The Enterobacteriaceae isolates were examined for carbapenemase production by MHT as CLSI recommendation [12]. An exactly 0.5 McFarland dilution of the E. coli ATCC 25922 in 5 ml of broth or saline was prepared. A 1:10 dilution was streaked as lawn on to a Mueller-Hinton agar plate. A 10 μg meropenem susceptibility disk was placed in the center of the test area. Test organism was streaked in a straight line from the edge of the disk to the edge of the plate. The plate was incubated overnight at 37°C for 16-24 hours. For quality control, K. pneumoniae ATCC1705 and K. pneumoniae ATCC1706 were served as a positive and negative control, respectively. After 24 hrs incubation, the positive test showed a cloverleaf-like indentation of the E. coli 25922 growing along the test organism growth streak within the disk diffusion zone, whereas, the negative test showed no growth of the E. coli 25922.

The study was approved by the research committee at the Faculty of Medical Laboratory Sciences, University of Khartoum, Sudan. This study was laboratory-based study and used bacterial isolates collected during routine microbiological investigations of clinical specimens and did not contain identifying information of the patients.

Statistical Analysis

SPSS program, version 16 (SPSS Inc., Chicago, IL) was used for data entry and analysis. Outcome data of isolates, their susceptibility patterns and the presences of ESBL, AmpC lactamase and carbapenemase-producing Enterobacteriaceae were tabulated in the form of proportions.

## Results

A total of 168 Enterobacteriaceae were isolated from 168 patients attending five referral hospitals in Khartoum state, Sudan. Of the 168 patients, 92 (54.8%) were females and the majority of them were adults (142, 84.5%). Most of the isolates were from urine sample (44%, 75), and wound swabs (44%, 75), followed by blood (9%, 15), body fluids (3%, 5) and ear swab (0.6%, 1). Among 168 isolates, 81 (47.4%) were E. coli, 52 (30.4%) were K. pneumoniae, 12 (7%) were Acinetobacter baumannii, 10 (5.8%) were Proteus mirabilis, eight (4.7%) were Enterobacter cloacae and five (2.9 %) were other Gram-negative bacteria.

Antimicrobial susceptibility

Table 1 summarizes the frequency of resistance rates of Enterobacteriaceae collected from patients at participated hospitals. Overall the isolates, the highest percentage of resistance rates were found in penicillins and cephalosporins antimicrobial classes. Good activity was shown to aminoglycosides and carbapenem.

**Table 1:** Antimicrobial resistant rates of Enterobacteriaceae (n = 168) collected from clinical specimens of patients at hospitals in Khartoum state, Sudan

Agent	E. coli (n = 81)	K. pneumoniae (n = 52)	A. baumannii (n = 12)	Proteus mirabilis (n = 10)	Enterobacter cloacae (n = 8)	Other Gram negative rods (n = 5)	Overall resistance (n = 168)
Amikacin	3.7 (3)	9.6 (5)	66.7 (8)	30 (3)	0.0 (8)	40 (2)	12.5 (21)
Augmentin	93.8 (76)	96.6 (50)	100 (12)	100 (10)	100 (8)	100 (5)	95.8 (161)
Amoxicillin	98.8 (80)	98.1 (51)	100 (12)	100 (10)	100 (8)	100 (5)	98.8 (166)
Ceftazidime	93.8 (76)	92.3 (48)	100 (12)	90 (9)	75 (6)	80 (4)	92.3 (157)
Ceftriaxone	91.4 (74)	94.2 (49)	100 (12)	90 (9)	75 (6)	4 (80)	91.7 (154)
Cefuroxime	96.3 (78)	96.0 (50)	100 (12)	90 (9.0)	100 (8.0)	100 (5.0)	96.4 (162)
Cefotaxime	93.8 (76)	92.3 (48)	100 (12)	90 (9.0)	62.5 (5)	80 (4)	91.7 (154)
Ciprofloxacin	76.5 (62)	61.5 (32)	100 (12)	80 (8)	50 (4.0)	80 (4.0)	72.6 (122)
Ofloxacin	76.5 (62)	63.5 (33)	100 (12)	80 (8.0)	50 (4.0)	80 (4.0)	73.2 (123)
Nitrofurantoin	22.2 (18)	57.7 (30)	91.7 (11)	80 (8.0)	50 (4.0)	100 (5.0)	45.2 (76)
Cefoxitin	38.3 (31)	40.4 (21)	100 (12)	100 (10)	100 (8.0)	60 (3.0)	44.6 (75)
Imipenem	7.4 (6.0)	34.6 (18)	75 (9.0)	10 (1)	37.5 (3.0)	40 (2.0)	23.2 (39)
Trimethoprim/sulfamethoxazole	75.3 (61)	75 (39)	100 (12)	90 (9.0)	50 (4.0)	100 (5.0)	77.4 (130)
Gentamicin	34.6 (28)	46.2 (24)	83.3 (10)	70 (7.0)	62.5 (5.0)	60 (3.0)	45.8 (77)

Amikacin and imipenem revealed good activities against most of the isolates except A. baumannii (66.7% and 75%, respectively). E. coli yielded high resistance rates for amoxicillin (98.8%), augmentin (93.8%), cefuroxime (96.3%), cefotaxime (93.8%), and ciprofloxacin (76.5%). Moderate resistance rates were observed among K. pneumoniae for ciprofloxacin (61.5%), ofloxacin (63.5%), nitrofurantoin (57.7%) and cefoxitin (40.4%).

ESBL and AmpC β lactamases production

**Figure 1:**
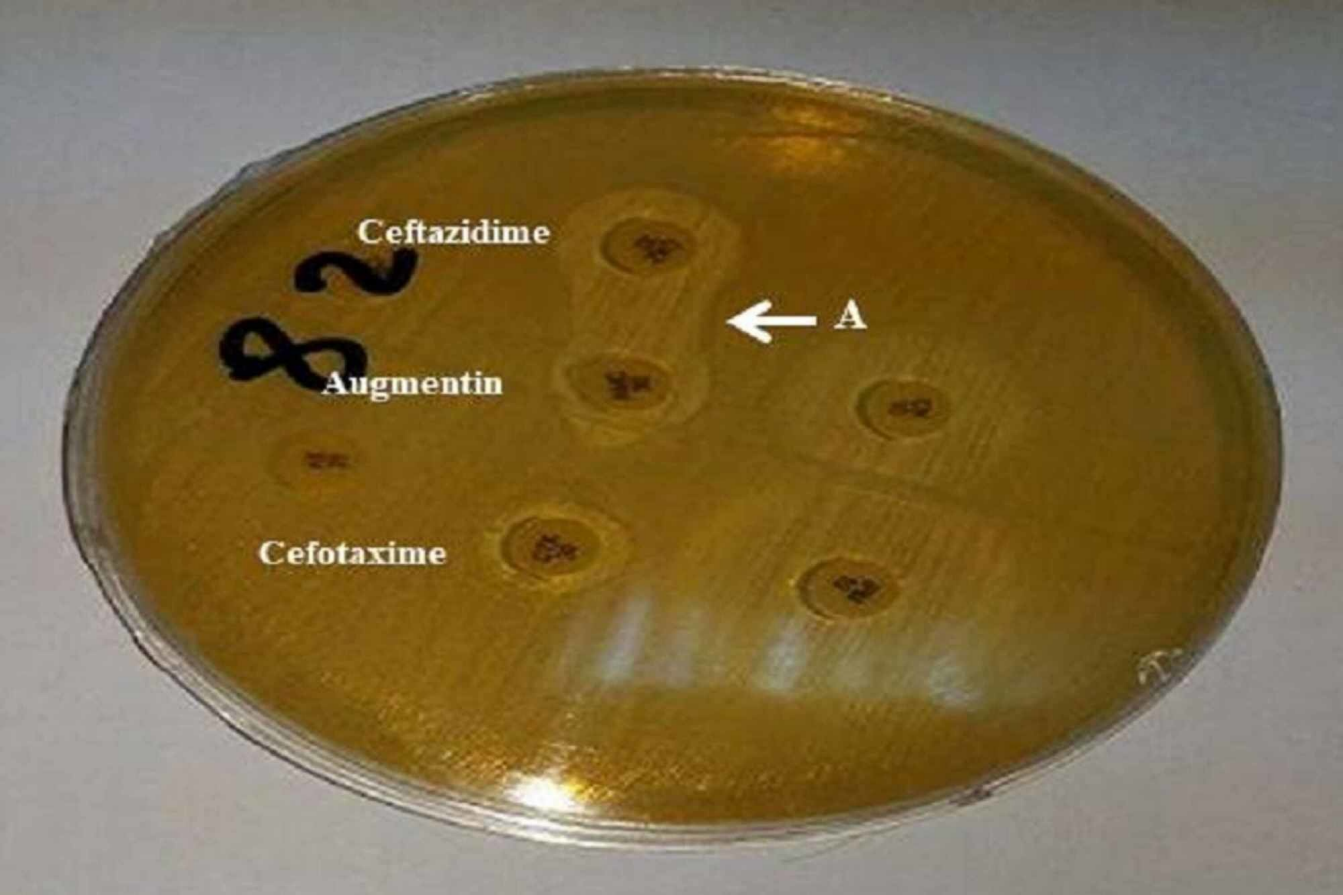
Double-disk synergy test showing positive result of ESBL production by Enterobacteriaceae, indicated by inhibition zone is enhanced (labeled A) between ceftazidime and clavulanic acid disks. ESBL: Extended-spectrum β-lactamase

Out of 168 Enterobacteriaceae screened for ESBL production, 45.2% (76) were positive indicated by enhancement of extended zone between amoxicillin clavulanic acid and cephalosporins (Figure 1). The highest frequency of ESBL enzymes was found among K. pneumoniae isolates (61.5%) (Table 2).

**Table 2:** Frequency of ESBL, AmpC β-lactamases and carbapenemase among Enterobacteriaceae members detected by different phenotypic laboratory methods. DDST: Double-disk synergy test; ESBL: Extended-spectrum β-lactamase; MHT: Modified Hodge test; ND: Not determined.

Isolates	ESBL	AmpC β-lactamases	Carbapenemase
	DDST	AmpC disk test	MHT
E. coli	37 (30/81)	29 (9/31)	67 (4/6)
K. pneumoniae	61.5 (30/52)	52.4 (11/21)	78 (14/18)
Acinetobacter baumannii	25 (3/9)	83.3 (10/12)	89 (8/9)
Proteus mirabilis	50 (5/10)	ND	0.0 (0/1)
Other Gram-negative rods	40 (2/5)	33.3 (1/3)	50 (1/2)
Enterobacter cloacae	50 (4/8)	75 (6/8)	67 (2/3)
Total	45.2 (76/168)	49.3 (37/75)	74.4 (29/39)

**Figure 2:**
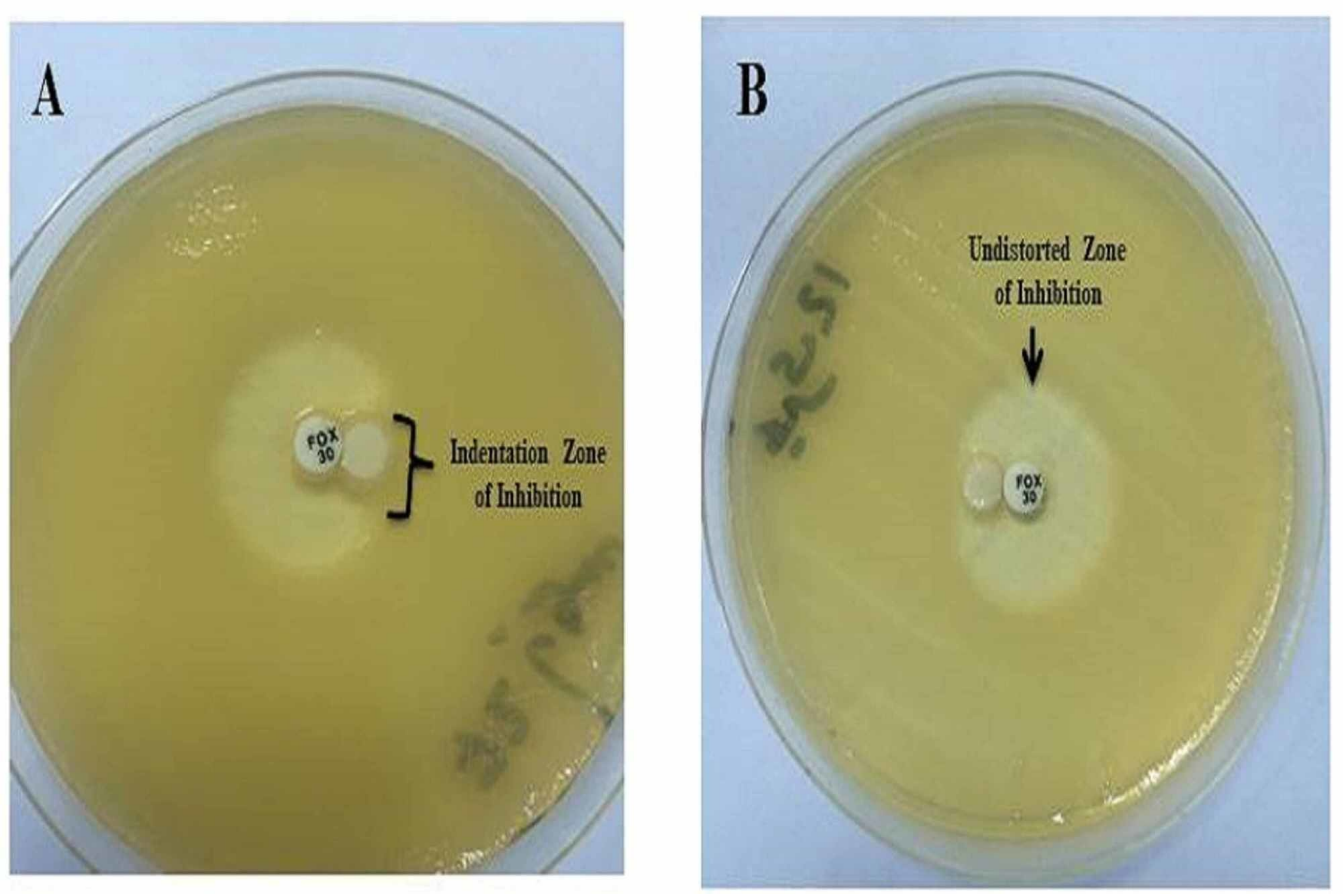
Plate A showing AmpC lactamase producing Enterobacteriaceae, indicated by the indentation of the cefoxitin zone of inhibition. Plate B showing negative AmpC isolates, indicated by an undistorted zone of inhibition.

Of the 75 cefoxitin insusceptible or showed an inhibition zone diameter of <18 mm and resistant to third generation cephalosporins, 49.3% were positive for AmpC β lactamases (Figure 2). A. baumannii was the common isolates carrying AmpC β lactamases (83.3%) followed by Enterobacter cloacae (75%) and K. pneumoniae (52.4%) (Table 2).

Carbapenemase production

**Figure 3:**
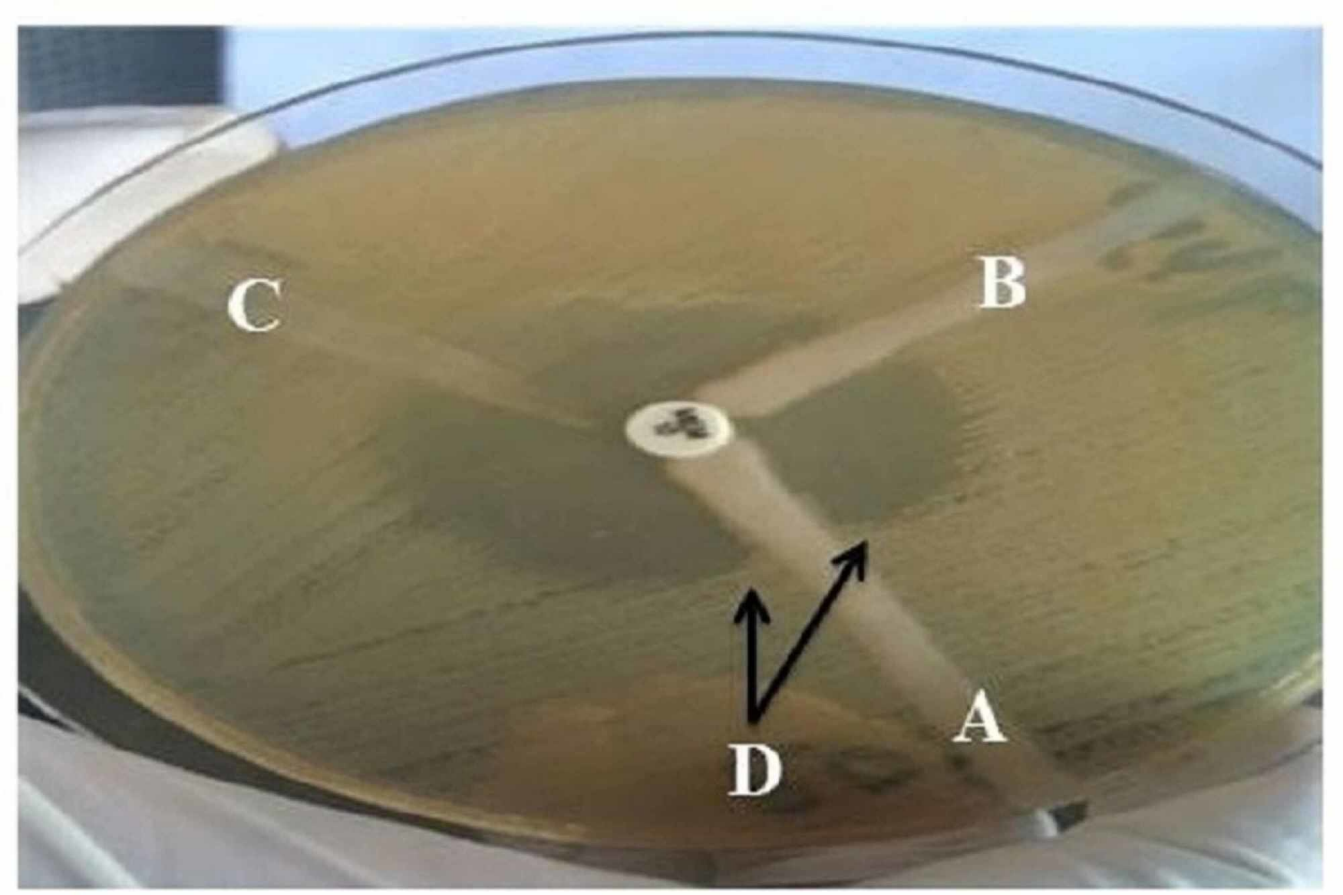
Modified Hodge test with meropenem disk (10 μg) for three Enterobacteriaceae isolates (A, B, C). Isolates labeled in A yielded positive result, indicated cloverleaf indentation (D).

Of the 39 isolates that yielded resistant or intermediate zone of diameter for imipenem disc, 29 (74.4%) were positive for carbapenemase production by MHT (Figure 3). The most frequent carbapenemase-producing organisms were A. baumannii (89%) and K. pneumoniae (78%) (Table 2).

## Discussion

Enterobacteriaceae isolates are increasingly spreading over the hospital settings and frequently responsible for most of the nosocomial and community-acquired infections [14]. In the present study, most of the isolates were recovered from urine samples and wound discharge. This finding in agreement with another result found that urinary tract infection (UTI) is a common disease among hospitalized patients [15]. In the present study, it is found that E. coli and K. pneumoniae are the leading Gram-negative pathogens. Similar results have been reported in Sudan, where E. coli and K. pneumoniae were the most frequent pathogens recovered from urine samples [16]. Likewise, a study in Uganda reported that K. pneumoniae (52%) and E. coli (44%) were the most frequent Gram-negative organisms from patients’ clinical specimens [17]. As well, global studies proved that infections caused by Gram-negative bacteria are predominant compared to other pathogens [18,19].

Infections caused by Enterobacteriaceae are usually treated by β-lactams antimicrobials of extended-spectrum cephalosporins and carbapenems groups [1]. In this study, most of the resistance was seen against penicillins, β-lactamase inhibitors, and cephalosporins groups (Table 1). Moreover, most of the pathogens revealed a high rate of resistance to the commonly used antimicrobial agents. A. baumannii demonstrated the higher resistance rates (100%) for cephalosporins and trimethoprim/sulfamethoxazole. However, K. pneumoniae, E. coli, P. mirabilis and other Enterobacteriaceae members showed variant high resistance rates to these agents. The high resistance rates reported here seemed to pose a significant concern in Khartoum state hospitals as trimethoprim/sulfamethoxazole and cephalosporins are the drug of choice for most of the Gram-negative infections. This escalating rate of resistance might attribute the presence of the ESBL or AmpC lactamases among the isolates. However, growing resistance to these antibiotics makes the rapid detection of such trait crucial for better therapeutic outcomes [1]. On the other hand, our Gram-negative isolates revealed good susceptibility for amikacin (12.3%) and imipenem (22.8%). Similar observations have been published by others [16,20,21]. For example, a recent study in Sudan found that imipenem is an effective agent against ESBL and non-ESBL Gram-negative bacteria from patients with urinary tract infections [16]. Furthermore, a study found that ESBL producing and non-producing Enterobacteriaceae remain susceptible to carbapenems, and these agents are considered preferred empiric therapy for serious Enterobacteriaceae infections [20].

ESBL, AmpC β lactamase, and carbapenemase are considered to be one of the most critical antibiotic resistance mechanisms in many Gram-negative pathogens [22]. In the present study, ESBL-producing Enterobacteriaceae were detected in 45.2% of isolates. This proportion was lower than those reported in Uganda (89%), and Brazil (61.1%), but relatively higher than 41% determined in the United Arab of Emirates [17,23,24]. It is well known that the use of expanded-spectrum cephalosporins in the hospital setting influences the selection of ESBL-producing organisms [25]. In this study, 61.5% of K. pneumoniae and 37% of E. coli found to be ESBL producers. Moreover, our finding revealed a high frequency of ESBL-producing Enterobacteriaceae, such as Proteus mirabilis and Enterobacter cloacae (Table 2). A previous study at Khartoum state hospitals found that ESBL producer was 30.2% among E. coli [26]. A similar observation has been documented elsewhere [17]. For instances, a study from Burkina Faso reported ESBL producers were more often found in E. coli (67.5%) and K. pneumoniae (26%) [8]. Another report from the Asia-Pacific region determined the highest frequency for ESBLs production was in E. coli (42.2%) followed by K. oxytoca (43.1%) and K. pneumoniae (35.8%) [2]. The occurrence of such high proportions of ESBL-producing microorganisms could be attributed to lack of antimicrobial surveillance program in healthcare settings. Ouedraogo et al. indicated that countries with limited resources where hygiene is poor and antibiotics are misused, the absence of anti-microbial surveillance programs increases the risk of multi-resistance development by bacteria in hospitals and the community [8].

Enterobacteriaceae-producing AmpC β-lactamases raise significant concerns as these microorganisms cause several nosocomial infections and high rate of treatment failure among infected patients [3]. It is well known that AmpC producers have the ability to explore multidrug resistance pattern to different types of antimicrobial classes [27]. In this study, 51.4% of isolates produce AmpC β lactamases, with peak levels among Acinetobacter baumannii, Enterobacter cloacae and K. pneumoniae. Similar observations have been reported in a previous study where AmpC β-lactamase was seen mainly in Acinetobacter spp (28.57%) followed by E. coli (6.97%) and Klebsiella spp. (6.18%) [28]. A study in India found that 80.9% of the Gram-negative isolates from a tertiary hospital were positive by AmpC Disc test, 47% were positive for plasmid-mediated AmpC beta-lactamases. Overall the isolates, 75% of Proteus were stable AmpC producers followed by Acinetobacter and Citrobacter 66.6% each, respectively [27]. Phenotypic methods for detection of AmpC β-lactamases have been proven to test provided a simple, convenient, and accurate means of detection of such resistant mechanisms [11]. Therefore, using cefoxitin insusceptibility test could be a useful diagnostic tool for detection of AmpC β-lactamases producing organisms which help in implementing an appropriate antimicrobial therapy and infection control strategies.

In the present study, 74.5% of the isolates were carbapenemase producers. The majority of them were A. baumannii (89%) and K. pneumoniae (78%). Carbapenemase-producing isolates have been increasingly reported in many parts of the world. A study in Iran showed that carbapenemase-producing organisms were E. coli (38%), followed by Pseudomonas aeruginosa (30%), K. pneumoniae (17%), Acinetobacter baumannii (12%), Citrobacter diversus (2%) and Enterobacter agglomerans (1.4%) [29]. Identification of carbapenemase is necessary for the surveillance of their transmission to hospitals and to overcome the problems associated with Gram-negative carbapenem resistance [7]. However, MHT is an easy and simple test to be performed to detect carbapenemases-producing bacteria [29].

## Conclusions

In conclusions, ESBL, AmpC β-lactamase and carbapenemase were commonly frequent in Enterobacteriaceae members that might increase the resistant patterns in our study area. Knowledge about the local epidemiology of such β-lactamases-producing bacterium is fundamental to establish an appropriate strategy for empirical therapy. The increasing prevalence of ESBL, AmpC-lactamase, and carbapenemase-producing Enterobacteriaceae necessitates reliable tests for detection and classification of such enzymes. However, simple phenotypic screening tests for routine detection of microorganisms harboring β-lactamase enzymes in hospital microbiology laboratories are recommended.
